# Vincamine Mitigates Methotrexate-Induced Liver Fibrosis Model

**DOI:** 10.5152/tjg.2025.24716

**Published:** 2025-06-23

**Authors:** Yonca Yılmaz Ürün, Gürkan Güner, Ejder Saylav Bora, Ayşe Buket Taşkın, Muslih Ürün, Oytun Erbaş

**Affiliations:** 1 Department of Gastroenterology, Yüzüncü Yıl University Medical School, Van, Türkiye; 2 Department of Medical Oncology, Medical Point Hospital, İzmir Economy University Faculty of Medicine, İzmir, Türkiye; 3 Department of Emergency Medicine, İzmir Atatürk Research and Training Hospital, İzmir, Türkiye; 4 Department of Internal Medicine, Medical Point Hospital, İzmir Economy University Faculty of Medicine, İzmir, Türkiye; 5 Department of Medical Oncology, Yüzüncü Yıl University Medical School, Van, Türkiye; 6 Department of Physiology, Demiroğlu Bilim University, İstanbul, Türkiye

**Keywords:** Liver fibrosis, methotrexate toxicity, oxidative stress, vincamine

## Abstract

**Background/Aims:**

Liver fibrosis is linked to higher rates of death and disease. This study examined the hepatoprotective properties of vincamine and its potential therapeutic application in treating liver damage caused by methotrexate in rats.

**Materials and Methods:**

Thirty male Wistar albino rats, with weights ranging from 150 to 200 g and ages between 10 and 12 weeks, were included in the study. A total of 10 rats were selected to serve as the control group, receiving no medication. A group of 20 rats was given a single intraperitoneal dose of 20 mg/kg methotrexate in order to cause liver damage. Subsequently, the participants were randomly allocated into 2 cohorts and administered either 1 mL/kg/day tap water or 50 mg/kg/day vincamine orally through gavage on a daily basis for a duration of 10 days. Following the completion of the treatment period, the animals were euthanized and their livers were examined histologically. Furthermore, the levels of plasma galectin-3 (gal-3), cytokeratin 18, malondialdehyde (MDA), alanine transaminase (ALT), liver MDA, and transforming growth factor beta (TGF-β) levels were evaluated. **Results: **Treatment with vincamine resulted in a significant decrease in plasma gal-3, cytokeratin, MDA, and ALT levels and liver MDA and TGF-β levels compared to the methotrexate and saline group. Vincamine treatment effectively protected against liver injury, and histopathological examination of the livers confirmed these results. **Conclusion: **This study demonstrates that vincamine alleviates methotrexate-induced liver toxicity via exhibiting antioxidant, anti-inflammatory, and anti-fibrotic activities and improved liver functionally, biochemically, and histopathologically.

**Main Points**

- Methotrexate, widely used for cancer, is associated with hepatotoxicity, leading to liver fibrosis through oxidative stress and inflammation.
- Vincamine demonstrates antioxidant, anti-inflammatory, and anti-fibrotic properties, showing promise as a protective agent against methotrexate-induced liver damage.
- This study underscores the potential of vincamine as a therapeutic agent to prevent methotrexate-induced liver fibrosis, warranting further investigation into its molecular mechanisms.

## Introduction

Liver fibrosis is a growing public health concern, marked by increasing prevalence and high mortality, particularly due to the rising prevalence of non-alcoholic fatty liver disease.^1^^,^^2^ It is characterized by the excessive accumulation of extracellular matrix (ECM), resulting in the distortion of the liver’s architecture due to the formation of fibrous scars. Its advancement to cirrhosis and hepatocellular carcinoma further highlights the need for effective treatment strategies. Despite scientific advancements, the etiology of liver fibrosis is not yet fully clarified, while oxidative stress and inflammation have been identified as key contributors to the initiation and progression of fibrogenesis.^3^^,^^4^ Importantly, reactive oxygen species (ROS) produced during liver injury—caused by factors such as viruses, drugs, and toxins—intensify liver damage and accelerate the progression of fibrosis.^5^

Liver fibrosis develops *via* a distinct adverse outcome pathway, characterized by a sequence of significant events: liver cell death or injury triggers the activation of Kupffer cells, which subsequently release the pro-fibrogenic cytokine transforming growth factor beta (TGF-β) to activate hepatic stellate cells (HSCs). Hepatic stellate cells play a crucial role in depositing ECM, leading to an abnormal accumulation of scar tissue in the liver. By inducing inflammation, apoptosis, and mitochondrial dysfunction, oxidative stress significantly contributes to the aggravation of liver injury.^6^

Methotrexate (MTX) has been in existence for approximately 70 years, functioning as a high-dose treatment (≥500 mg/m^2^) for cancer and a low-dose plan (5-25 mg/week) for the management of autoimmune conditions such as psoriasis or rheumatoid arthritis.^7^^,8^ Even though the effectiveness and tolerance of MTX are widely recognized, there has consistently been apprehension regarding potential liver toxicity.^9^ Methotrexate-induced hepatic injury is marked by oxidative stress, disrupted mitochondrial respiration, and endoplasmic reticulum stress. These mechanisms play a role in liver fibrosis development via 2 separate pathways.^10^

Liver fibrosis development has been linked to the accumulation of methotrexate and its metabolites. Methotrexate-induced hepatotoxicity is driven by impaired DNA synthesis, causing folic acid depletion and subsequent hepatocellular injury. In parallel, MTX induces HSC activation, fostering oxidative stress, inflammatory responses, and apoptosis in hepatocytes. These combined mechanisms culminate in liver damage, as indicated by elevated transaminases, inflammatory biomarkers, and increased liver stiffness, which serve as early indicators of the onset of liver fibrosis.^11^ If left untreated, liver fibrosis can advance to liver cirrhosis, potentially causing hepatic insufficiency, portal hypertension, and, in severe cases, liver failure.^10^

Vincamine functions as a peripherally acting vasodilator, enhancing cerebral blood flow and glucose uptake, and is commonly used as a nootropic agent to counteract age-related cognitive decline.^12^^,^^13^ Beyond its neurovascular effects, vincamine has been shown to exhibit potent antioxidant, anti-inflammatory, and anti-apoptotic properties, which are relevant to the pathogenesis of liver fibrosis.^14^ Recent experimental studies have highlighted vincamine’s potential to attenuate tissue injury by reducing oxidative stress, modulating cytokine expression, and preserving mitochondrial function.^15^^,^^16^ These mechanisms are particularly important in the context of hepatic fibrogenesis, where oxidative damage, inflammation, and apoptosis contribute to the activation of HSCs and excessive ECM deposition. However, the potential anti-fibrotic effects of vincamine in the liver remain largely unexplored.

The primary objective of this study was to investigate the hepatoprotective and anti-fibrotic effects of vincamine in a rat model of methotrexate-induced liver injury. This research aimed to evaluate the impact of vincamine on oxidative stress, inflammation, and fibrotic markers to provide insights into its mechanisms of action and therapeutic potential.

## Materials and Methods

### Animals

This study involved the use of 30 male Wistar albino rats, with a weight range of 150-200 g and an age range of 10-12 weeks. The experiments conducted followed the guidelines specified in the “Guide for the Care and Use of Laboratory Animals” established by the National Institutes of Health (USA). The study was conducted in accordance with the Declaration of Helsinki and approved by the Institutional Animal Care and Ethical Committee at the Bilim University, identified by the ethical reference number 2223103904 (Date: August 8, 2022). The experimental rats were obtained from the Experimental Animal Laboratory of Bilim University. The rats were given unrestricted access to food, kept in steel cages in a temperature-controlled environment (22°C ± 2°C), and exposed to a 12-hour light/darkness cycle.

### Experimental Protocol

The study comprised a cohort of 30 male rats. Out of the group, 20 rats were administered a single intraperitoneal dose of 20 mg/kg methotrexate to induce liver damage. The remaining 10 rats comprised the control group and were not subjected to any chemical administration. The 20 rats that received methotrexate were subsequently split into 2 groups. Group 1 rats were given vincamine orally at a dosage of 50 mg/kg/day using a gavage technique, while Group 2 rats were orally administered 1 mL/kg/day of tap water *via* gavage. The administration of all treatments spanned a duration of 10 days. Following the study, euthanasia was carried out on all animals by means of cervical dislocation while under anesthesia induced by administering 100 mg/kg of ketamine (Ketasol, Richter Pharma AG, Austria) and 50 mg/kg of xylazine (Rompun, Bayer, Germany). A cardiac puncture was performed to collect blood samples for subsequent biochemical analysis. The liver was removed for histopathological and biochemical analyses.

### Histopathological Evaluation

After the animals were sacrificed, liver tissues were promptly excised and fixed in 10% neutral buffered formalin for at least 24 hours. Following fixation, the tissues were dehydrated through a graded ethanol series, cleared in xylene, and embedded in paraffin blocks. Sections of 4 μm thickness were obtained using a microtome and mounted on glass slides. These sections were stained with hematoxylin and eosin to evaluate general liver architecture and cellular morphology.

Histopathological evaluation was performed under light microscopy using an Olympus BX51 microscope (Olympus Corp., Tokyo, Japan), and representative images were captured with an Olympus C-5050 digital camera. The evaluation and scoring of liver injury were conducted in a blinded manner by an experienced histopathologist.

Liver damage was assessed semi-quantitatively based on the method described by Lobenhofer et al.^17^ The scoring system included the following parameters: hepatocyte necrosis, inflammatory cell infiltration, and fibrosis. Each parameter was graded on a scale from 0 to 4, defined as follows: 0 = No change (normal histology), 1 = Minimal (focal changes affecting <10% of the section), 2 = Mild (changes affecting 10%-25% of the section), 3 = Moderate (changes affecting 26%-50% of the section), 4 = Marked (diffuse or confluent changes affecting >50% of the section). Percentages were calculated using IMAGE J. The total liver injury score was calculated by summing the individual scores of these parameters for each animal. Higher cumulative scores indicated more severe hepatic damage. The results were statistically analyzed to determine differences between experimental groups.

### Liver Biochemical Analysis

After the removal of the head, the livers were immediately removed and kept at a temperature of −20 °C until they were prepared for biochemical analysis. To perform tissue analysis, liver tissues were homogenized using a glass homogenizer in a solution of phosphate-buffered saline (pH 7.4) at a ratio of 5 parts solution to 1 part tissue. The resulting mixture was then subjected to centrifugation at a force of 5000 times the acceleration due to gravity for a duration of 15 minutes. The supernatant obtained was collected, and the protein concentration in the liver homogenates was determined using Bradford’s method, with bovine serum albumin as the reference standard.^18^

The TGF-β concentrations in the liver supernatants were measured using commercially available rat enzyme-linked immunosorbent assay (ELISA) kits from R&D Systems, Houston, TX, USA. Replicate measurements for all samples from each animal were performed in accordance with the instructions provided by the manufacturer. The absorbance values were quantified using a microplate reader (MultiscanGo, Thermo Fisher Scientific Laboratory Equipment, NH, USA).

### Measurement of Plasma Galectin-3 and Cytokeratin 18 Levels

The levels of galectin-3 and cytokeratin 18 in plasma were measured using ELISA kits obtained from R&D Systems in Houston, TX, USA.

### Determination of Lipid Peroxidation

To assess lipid peroxidation, the concentrations of malondialdehyde (MDA) were measured as thiobarbituric acid reactive substances (TBARS) in both tissue and plasma samples.^19^ The tissue samples were treated with trichloroacetic acid and TBARS reagent, mixed together, and then incubated at a temperature of 100°C for 60 minutes. The samples were cooled on ice and then centrifuged at a speed of 3,000 revolutions per minute for 20 minutes. The optical density of the liquid remaining after centrifugation, referred to as the supernatant, was quantified at a wavelength of 535 nanometers. The tissue’s MDA levels were quantified using tetraethoxypropane and a standard calibration curve. The results were quantified in terms of nmol/g of protein.

### Determination of Plasma Alanine Aminotransferase Levels

The plasma levels of alanine aminotransferase (ALT) were measured using an ELISA kit obtained from R&D Systems, a company based in Houston, TX, USA.

### Statistical Analysis

Statistical analyses were performed using SPSS software version 22 (IBM SPSS Corp.; Armonk, NY, USA). The variables were investigated using visual (histograms, probability plots) and analytical methods (Kolmogorov-Simirnov/Shapiro-Wilk’s test) to determine whether or not they are normally distributed. One-way ANOVA was used when comparing more than 2 groups because the data were normally distributed. Levene test was used to assess the homogeneity of the variances. When an overall significance was observed, pairwise post-hoc tests were performed using Tukey’s test. The *P*-values obtained in the post-hoc analysis were corrected with Bonferroni correction. The data are presented as the mean ± standard error of the mean (SEM) and statistical significance was assessed at *P* values of .05 or below.

## Results

### Plasma and Liver Malondialdehyde Levels

The impact of vincamine on oxidative stress caused by methotrexate was evaluated by measuring plasma and liver malondialdehyde levels, which are indicators of lipid peroxidation. The levels of plasma and liver MDA were significantly higher in rats administered with methotrexate and saline, compared to the control group (*P* < .001 and *P* < .01, respectively). The levels of MDA in the plasma and liver of rats that were administered methotrexate and vincamine were significantly lower compared to the group that received methotrexate and saline (*P* < .05 and *P* < .001, respectively) (Table 1).

### Plasma Alanine Aminotransferase, Galectin-3, Cytokeratin 18 Levels, and Liver TGF-β Levels

The assessment of alterations in cytokine expression was conducted to evaluate the inflammatory response caused by methotrexate and the beneficial impact of vincamine. The administration of methotrexate and saline resulted in an increase in the expression of liver TGF-β, which is a key cytokine involved in fibrosis and a strong activator of HSCs. This increase was observed when compared to the control group, with statistical significance at a *P*-value of less than .01. Vincamine treatment improved the levels of TGF-β in rats that were given methotrexate (*P* < .05) (Table 1).

The levels of galectin-3 in the plasma were significantly higher in rats treated with methotrexate and saline compared to the control group (*P* < .01). The administration of vincamine led to a notable reduction in plasma galectin-3 levels in comparison to the methotrexate and saline group (*P* < .001) (Table 1). The levels of plasma cytokeratin 18 were significantly higher in rats treated with methotrexate and saline compared to the control group (*P* < .01). Administration of vincamine led to a notable reduction in plasma cytokeratin 18 levels compared to the methotrexate and saline group (*P* < .001) (Table 1).

The plasma alanine aminotransferase (ALT) levels were significantly higher in rats administered with methotrexate and saline compared to the control group (*P* < .01). Rats treated with vincamine exhibited a notable decrease in plasma ALT levels compared to the methotrexate-saline group (*P* < .05) (Table 1).

### Histopathological Examination of Liver Tissue Samples

**Figure 1.**
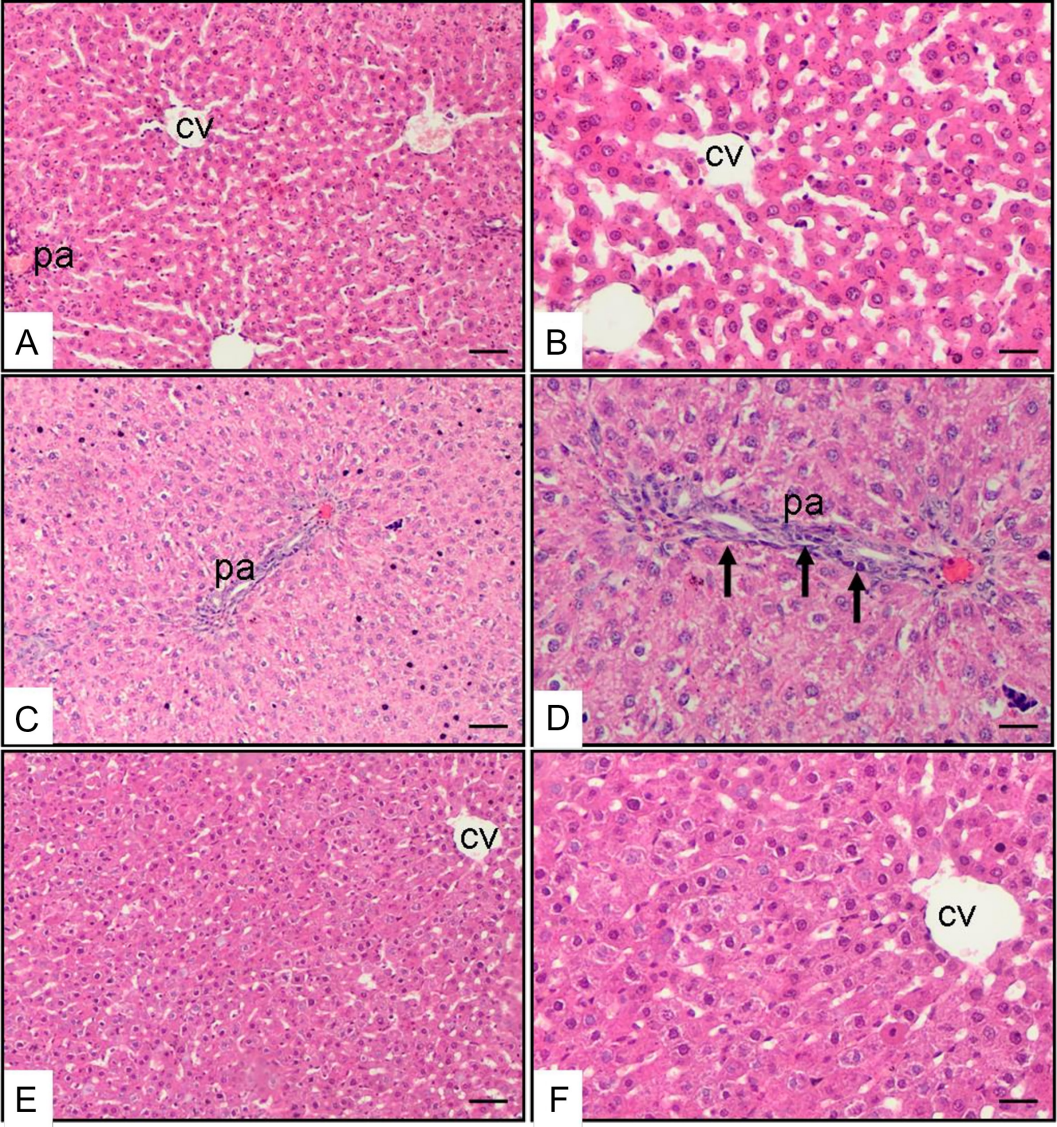
Liver histopathology Hematoxylin and eosin stain (A, C, E ×10 and B, D, F ×20 magnification, scale bar: 100 µm), A-B: Normal group rats have normal livers. Central vein (CV), C-D: Methotrexate and saline group rats have bridging necrosis, fibrosis, and cellular infiltration in the portal area (pa) (arrow), E-F: Methotrexate+Vincamine group rats have decreased bridging necrosis, fibrosis, and cellular infiltration.

The histopathological examination of the livers of control and methotrexate and saline-treated rats revealed that the livers from the methotrexate and saline group show cellular infiltration (*P* < .01), hepatocyte necrosis (*P* < .001), and fibrosis (*P* < .01). Vincamine provided significant protection and prevented liver fibrosis induced by methotrexate. In the methotrexate and vincamine group, there was a decrease in cellular infiltration (*P* < .001), hepatocyte necrosis (*P* < .05), and fibrosis (*P* < .001) in the livers compared to the methotrexate and saline group (Table 2). The histopathological features of the livers are shown in Figure 1.

## Discussion

Methotrexate treatment is strongly associated with hepatotoxicity, which is a highly significant and serious adverse outcome. Alleviating this adverse effect not only improves the welfare of the patient but also contributes to the overall efficacy of the treatment. This study is the first to examine the effects of vincamine treatment on methotrexate-induced liver damage in rats.

The study findings revealed that vincamine exhibited significant hepatoprotective effects and effectively alleviated methotrexate-induced liver fibrosis. Methotrexate administration led to marked histological changes, such as hepatocyte necrosis, fibrosis, and increased cellular infiltration. The findings are consistent with prior studies.^20^^,^^21^^,^^22^ The liver sections of the control group exhibit a characteristic histological morphology. The histological appearance of liver sections in the group treated with both MTX and vincamine shows a significant improvement compared to the group treated with MTX and saline. In addition, a correlation between these histological changes and the results of serum and biochemical studies was established, which also provided evidence of liver injury.

Numerous studies, involving both patients and animals, as well as in *vitro* investigations, have been undertaken to explore various pathways associated with MTX hepatotoxicity. These studies reveal that the retention and buildup of MTX polyglutamates, coupled with the resulting depletion of folic acid in liver cells, contribute to an increase in liver enzyme levels. This phenomenon may, in part, be accountable for the hepatotoxic effects induced by MTX.^23^ The extent of hepatic injury resulting from MTX intoxication was assessed through the measurement of the transaminase ALT levels. The increase in ALT levels has been linked to impaired structural integrity in the liver, as these enzymes are normally found in the cytoplasm and are released into the bloodstream after damage to the cells. The observations unequivocally illustrated that MTX treatment leads to an elevation in serum ALT levels, consistent with findings from previous studies.^20^^,^^21^^,^^22^

Mounting evidence suggests a connection between tissue injury induced by anticancer drugs and the generation of free radicals, leading to oxidative stress.^24^ Given the established role of oxidative stress in contributing to MTX-induced liver fibrosis,^25^ the regulatory impact of vincamine on MTX-induced oxidative stress was explored by evaluating levels of MDA in both plasma and the liver. This study found a notable increase in both plasma and liver MDA levels in rats treated with MTX and saline, compared to the normal group, indicating lipid peroxidation in the liver. The increased lipid peroxidation can be ascribed to the production of ROS triggered by MTX, resulting in a reduction in cellular antioxidant levels.^25^ These findings further support the role of oxidative stress in MTX-induced liver fibrosis. Notably, the administration of vincamine to rats was observed to significantly alleviate the lipid peroxidation induced by MTX. This can be attributed to its previously confirmed antioxidant properties.^15^^,^^16^ Consequently, vincamine possesses the potential to reinstate cellular defense mechanisms and impede lipid peroxidation.

Within the scope of this research, the proposal was that the application of MTX led to an increase in serum TGF-β levels, indicating the participation of this cytokine in MTX-induced liver damage. Transforming growth factor-beta is a key cytokine that promotes fibrosis and strongly activates HSCs. Transforming growth factor-beta, a vital regulator in chronic liver disease, has a significant impact on every stage of disease progression, beginning with the initial liver injury and leading to the formation of fibrosis.^26^ This observation indicates an increase in the number of activated hepatocellular stellate cells that are expressing TGF-β. Prior studies have demonstrated that inhibiting the TGF-β signaling pathway can mitigate liver fibrosis.^22^^,^^27^ These findings align with this notion, as vincamine administration resulted in the downregulation of TGF-β, indicating the anti-fibrotic potential of vincamine. Earlier experimental investigations have additionally noted the anti-fibrotic properties of vincamine, as evidenced by a reduction in fibrotic markers observed in pulmonary fibrosis,^28^ cardiac fibrosis,^29^ and liver fibrosis.^30^

An additional noteworthy discovery from the study is that exposure to MTX resulted in an elevation of plasma galectin-3, a glycoprotein belonging to the galectin family. Galectin-3 is directly involved in liver fibrosis by activating macrophages and myofibroblasts through the TGFβ pathway,^31^ making galectin-3 levels a reliable biomarker for liver diseases.^32^ The utilization of galectin-3 as a marker for MTX-induced hepatotoxicity was suggested, with vincamine treatment demonstrating the capability to decrease plasma galectin-3 levels. Cytokeratin 18, constituting 5% of hepatic proteins, serves as a substantial component in the liver and has been recognized as a promising biomarker for drug-induced liver injury.^33^ This experiment revealed a notable reduction in plasma levels of cytokeratin 18 in the group treated with both methotrexate and vincamine, as opposed to rats treated with methotrexate and saline. These results align with the existing literature.

This study demonstrates that vincamine exerts significant hepatoprotective and anti-fibrotic effects against methotrexate-induced liver injury in rats, primarily through its antioxidant, anti-inflammatory, and anti-apoptotic properties. By reducing oxidative stress, downregulating pro-fibrotic markers such as TGF-β and galectin-3, and limiting hepatocyte apoptosis, vincamine effectively mitigated liver damage and fibrosis, as supported by both histopathological and biochemical findings. These results highlight the potential of vincamine as a therapeutic candidate for the prevention or attenuation of drug-induced liver fibrosis. However, to support its clinical applicability, further studies are needed to validate these effects, clarify the underlying molecular mechanisms, assess long-term safety, and determine optimal dosing. If confirmed in future clinical trials, vincamine may offer a novel, accessible option for managing methotrexate-induced hepatotoxicity and possibly other forms of liver fibrosis.

## Figures and Tables

**Table 1.** Effect of Vincamine on Biochemical Analysis Results Related to Methotrexate-Induced Liver

	Normal	MTX and Saline	MTX and Vincamine
Liver TGF-β level (pg/g)	0.8 ± 0.05	1.6 ± 0.08*	1.06 ± 0.04#
Plasma galectin-3 level (pg/mL)	164.2 ± 7.3	438.5 ± 7.7*	265.1 ± 4.9##
Plasma cytokeratin 18 level (pg/mL)	0.69 ± 0.2	3.04 ± 0.5*	1.1 ± 0.2##
Plasma MDA level (nM)	52.7 ± 1.6	143.7 ± 10.1**	96.5 ± 8.6#
Liver MDA level (nmol/g tissue)	29.2 ± 0.9	77.8 ± 2.5*	41.03 ± 2.3##
ALT (IU/L)	18.2 ± 1.6	57.1 ± 3.4*	33.5 ± 1.9#

Results are presented as mean ± SEM. Statistical analyses were performed by one-way ANOVA test.

ALT, alanine aminotransferase; MDA, malondialdehyde; MTX, methotrexate; nanomole/gram; nM, nanometer; nmol/g; pg/g, picogram/gram; pg/mg, picogram/milligram; pg/mL, picogram/milliliter; TGF-β, transforming growth factor beta.

*P < .01.

**P < .001 (different from normal group).

#*P* < .05.

##*P* < .001 (different from MTX and saline group).

**Table 2.** Comparison of Groups According to the Liver Histopathological Scoring System

	Normal	MTX and Saline	MTX and Vincamine
Hepatocyte necrosis	0.1 ± 0.1	1.8 ± 0.4 **	1.2 ± 0.1#
Fibrosis	0.2 ± 0.1	2.1 ± 0.2 *	0.5 ± 0.2##
Cellular infiltration	0.1 ± 0.1	1.1 ± 0.1 *	0.4 ± 0.1##

Results are presented as mean ± SEM. Statistical analyses were performed by one-way ANOVA test.

MTX, methotrexate.

*P < .01.

**P < .001 (different from normal group).

#*P* < .05.

##*P* < .001 (different from MTX and saline group).

## Data Availability

The data that support the findings of this study are available on request from the corresponding author.
